# Supplementary material for: Molecular acclimation of Halobacterium salinarum to halite brine inclusions
Source: Front Microbiol. 2023 Jan 26;13:1075274. doi: 10.3389/fmicb.2022.1075274 (PMC9976938; doi:10.3389/fmicb.2022.1075274)
Supplement: Supplementary file 6 [file Data_Sheet_1.PDF]

Supplementary Information

1 Supplementary Section 1: Halite Surface Contaminant Removal.

Supplementary Table 1-1: Summary of previously published protocols for the deactivation of microorganisms and removal of DNA from halite surfaces. It is important to note that none of these protocols were intended (or tested) for removal of proteins contaminating halite surfaces.

Article	Dombrowski 1963<br>Radax 2001<br>Gruber 2003	Norton & Grant 1988<br>Norton, McGenity, Grant 1993	Stan-Lotter 1993<br>Denner 1994	Rosenzweig 2000		Gramain 2011			Sankaranarayanan 2011	
Samples		natural halite		laboratory-grown halite: 96well plate naturally contaminated at the surface by an "unidentified white halophile"		laboratory-grown halite (tested by coating halite surface with *Halococcus saccharoluticus* and/or *Halobacterium salinarum*)			natural halite from saline Valley California (immersion volume = 4 mL indicating relatively small crystal size) (tested by spiking halite surface with DNA; HV1 region of human mitochondrion)	
Targets		cell lysis		cell lysis (confirmed by CFU counts)		cell lysis (confirmed by growth test) & DNA removal			DNA removal	
Solutions	NaCl unsatured	NaCl unsatured	NaCl unsatured	NaCl-saturated except for HCl		NaCl-saturated except for HCl			NaCl-saturated except for HCl	
Treatment & steps	Bunsen burner	EtOH 100% -- 24h	EtOH 100% -- 6h	* 2 % SDS -- 1 min<br>* 0.02 % TA -- 1 min<br>———<br>* 2 % SDS -- 1 min<br>* 0.02 % TA -- 1 min<br>*10 M NaOH -- 1 min<br>———<br>* 2 % SDS -- 1 min<br>* 0.02 % TA -- 1 min<br>* 10 M NaOH -- 1min<br>* 10 N HCl -- 1 min	* 10 M NaOH -- 5 min<br>* saturated NaCl -- 2 min<br>* 10 N HCl -- 5 min<br>* brine saturated Na2CO3 -- 2 min	**A-**<br>* 10 M NaOH -- 5 min<br>* saturated NaCl -- 2 min<br>* 10 N HCl -- 5 min<br>* brine saturated Na2CO3 -- 2 min<br>**B-**<br>* 2 % SDS -- 1 min<br>* saturated NaCl -- 1 min<br>* 0.02 % TA -- 1 min<br>* saturated NaCl -- 1 min<br>* 10 M NaOH -- 1min<br>* saturated NaCl -- 1 min<br>* 10 N HCl -- 1 min	**C-**<br>* 70 % EtOH -- 1 min<br>* saturated NaCl -- 1 min<br>* 6 % NaOCl -- 1 min<br>* saturated NaCl -- 1 min<br>**D-**<br>* 70 % EtOH -- 3 min<br>* saturated NaCl -- 2 min<br>* 6 % NaOCl -- 3 min<br>* saturated NaCl -- 2 min<br>* 10 M NaOH -- 3 min<br>* saturated NaCl -- 2 min<br>* 10 N HCl -- 3 min<br>* saturated Na_2CO_3/NaCl -- 2 min	**E-**<br>* 6 % NaOCl -- 20 min<br>* saturated NaCl -- 5 min<br>* 10 M NaOH -- 20 min<br>* saturated NaCl -- 5 min<br>* 10 N HCl -- 20 min<br>* saturated Na2CO3/NaCl -- 20 min<br>* saturated NaCl -- 5 min<br>* 70 % EtOH -- 20 min<br>* saturated NaCl -- 5 min	# Al<br># Al-Bl<br># Al-Ac<br># Ac<br># Bl<br># Et<br># Bl-Et<br># Ac-Bl-Et<br># Ac-Et	# Al-Ac-Bl<br>* 10 M NaOH -- 15 min<br>* saturated NaCl -- 15 min<br>* 10 N HCl -- 15 min<br>* Na_2CO_3 -- 15 min<br>*saturated NaCl -- 15 min<br>* 6 % NaOCl -- 15 min<br>* saturated NaCl -- 15 min (x4)<br># Ac-Bl<br>* 10 N HCl -- 15 min<br>* Na_2CO_3 -- 15 min<br>* saturated NaCl -- 15 min<br>* 6 % NaOCl -- 15 min<br>* saturated NaCl -- 15 min (x4)
Conclusions	used	used	used	rejected	validated	rejected	rejected	validated	rejected	validated

2 Supplementary Section 2: Halophile Protein Extraction Protocol Summary for Bench Top Use

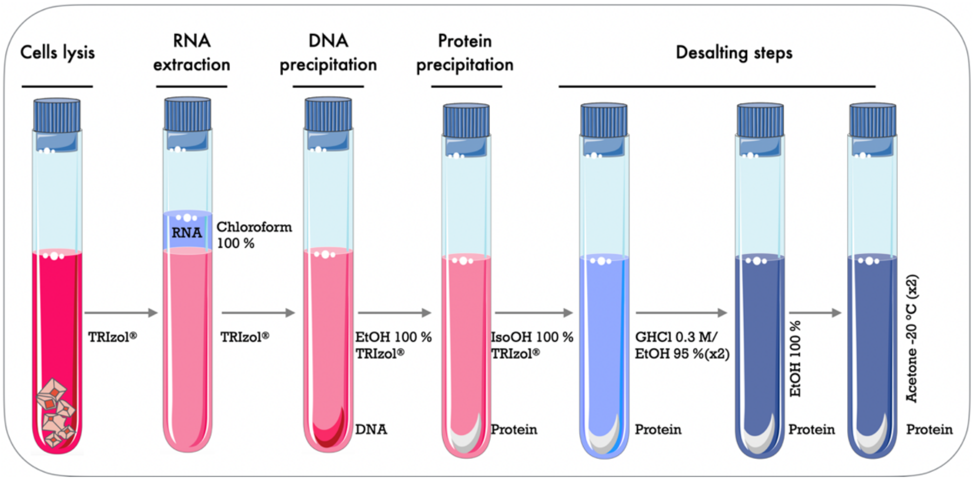

Supplementary Figure 2-1: Schematic overview of sequential extraction using TRIzol reagent. EtOH, IsoOH and GHCl abbreviations were used for ethanol, isopropanol and guanidine-HCl respectively.

Bench Top Protocol for Halophile Protein Extraction

For all steps, sterilize solutions and materials by autoclave. All reagents should be analytical and HPLC grade. The use of glass materials including autoclaved glass Pasteur pipets is strongly recommended to avoid potential organic contamination from plastic polymers.

Cell Collection and Lysis

1. Sample preparation:
    A. **For liquid extraction:** Add sufficient volume of culture for 2 x 10^{10} cells to a glass centrifuge tube. Centrifuge 7 500 x g / 10 min / room temperature and discard supernatant.
    B. **For extraction from salt crystals:** Directly after removal of salt crystal surface bound contaminants using the NaOCl-NaOH-HCl active-spray surface organics method, add crystals to a glass centrifuge tube.
2. Add 5 mL of TRIzol reagent™ in each tube, ensure that crystals were fully immersed.
3. Crush crystal or cell pellet using autoclaved glass rod for approximately 5 min. Verify the absence of residual crystal fragments.
4. Vortex gently 30 s; incubate at 65 °C for 20 min.
5. Cooling tubes 5 min at room temperature (RT).

RNA Fraction Removal

6. Add 1 mL of 100 % chloroform in tube and parafilm.
7. Vortex gently 30 s; incubate at 5 min, RT
8. Centrifuge 10 000 x g, 20 min, 4 °C and discard very carefully upper colorless phase and dense middle phase.

DNA Precipitation

9. Add 1.5 mL of 100 % ethanol in tube and parafilm.
10. Vortex 3 s and incubate 3 min, RT.
11. Centrifuge 2 000 x g, 10 min, 4 °C
12. Collect carefully supernatant in a new 30 mL centrifuge tube.

Protein Precipitation

13. Add 7.5 mL 100 % isopropanol in tube and parafilm. Do not mix by vortex to avoid proteins adhering to the centrifuge tube wall.
14. Incubate 15 min, RT.
15. Centrifuge 10 000 x g, 20 min, 4 °C and very carefully discard supernatant.

Protein Pellet Desalting and Phenol Removal

16. Add 5 mL of 0.3 M Guanidine-HCl in 95 % ethanol. Do not mix by vortex to avoid proteins adhering to the centrifuge tube wall.
17. Incubate 20 min, RT. If necessary, samples can be temporarily stored at -20°C before proceeding to the next step
18. Centrifuge 10 000 x g, 20 min, 4 °C and carefully discard supernatant.
19. Repeat steps 16-18.
20. Add 5 mL of 100 % ethanol, parafilm.
21. Incubate 20 min, RT.
22. Centrifuge 10 000 x g, 20 min, 4 °C and carefully discard supernatant.
23. Add 2 mL 100 % glacial acetone (-20 °C), parafilm.
24. Incubate 1h at -20 °C
25. Centrifuge 10 000 x g, 20 min, 4 °C and carefully discard supernatant.
26. Repeat steps 23-25.
27. Air drying protein pellet under hood (opened tubes in ice) until complete acetone evaporation.

Protein Resuspension

28. Gradually resuspend protein pellet with 2 mL 1 M $NaHCO_3$, 0.1 % sodium dodecylsulfate (SDS) at RT over 2 days. If the solution remains turbid, add more resuspension buffer.

Protein Quantification Assay

29. Quantitate resuspended proteins using the Pierce® BCA kit according manufacturer's instructions. After assay, aliquot proteins and stock at -20 °C for further analysis.

Proteins Digestion

30. Using a 100 µg aliquot of proteins from the previous step, reduced di-sulfur bonds using 2 mM of tris-(2-carboxyethyl) phosphine (TCEP) for 1h at 37 °C.
31. Alkylate cysteines with 5 mM iodoacetamide for 30 min at RT in the dark.
32. Digest proteins with 5 µg of Trypsin Gold (Promega) for 15 h at 37 °C with continuous agitation

Peptide Desalting by Solid Phase Extraction (SPE)

33. Use Sep-Pak C18 Plus cartridge Short 400 mg Sorbent (Waters) and elute peptides with 2 mL 70 % acetonitrile, 30 % (H_2O, 0.1 formic acid).
34. SpeedVac eluted peptides and resuspend them in 50 µL of 0.5 M TEAB

Isobaric Labelling of Peptides

35. Using 50 µg of initial proteins, labelled peptides with 8-plex iTRAQ® according manufacturer's instructions. Then desalting labelled peptides with SPE by repeating steps 33-34.

Resuspend labelled peptides in 2 % acetonitrile, 98 % (H2O, 0.1 % formic acid) using the appropriate volume for the desired peptide concentration. Stock at -20 °C until mass spectrometry analysis.

3 Supplementary Section 3: Protein Quantitation Using Modified BCA Assay.

Standard BCA protein assay allowed quantitation of protein concentrations between 125 and 2 000 µg with a microplate procedure using 1:20 sample:working reagent ratio. This protocol does not allow for detection of allowing low protein concentrations such as those obtained after halite surface cleaning. We therefore used modified concentrations of bovine serum albumin (BSA) standards for a working range between 25 and 200 µg proteins (see Supplementary Table 3-1), to establish the protein standard curve presented in supplementary Figure 3-1.

Supplementary Table 3-1: BSA standards concentrations for standard BCA and modified BCA assay.

Standard procedure BSA concentration (µg/mL)	Modified procedure BSA concentration (µg/mL)
2 000	200
1 500	175
1 000	150
750	125
500	100
250	75
125	50
25	25
0	0

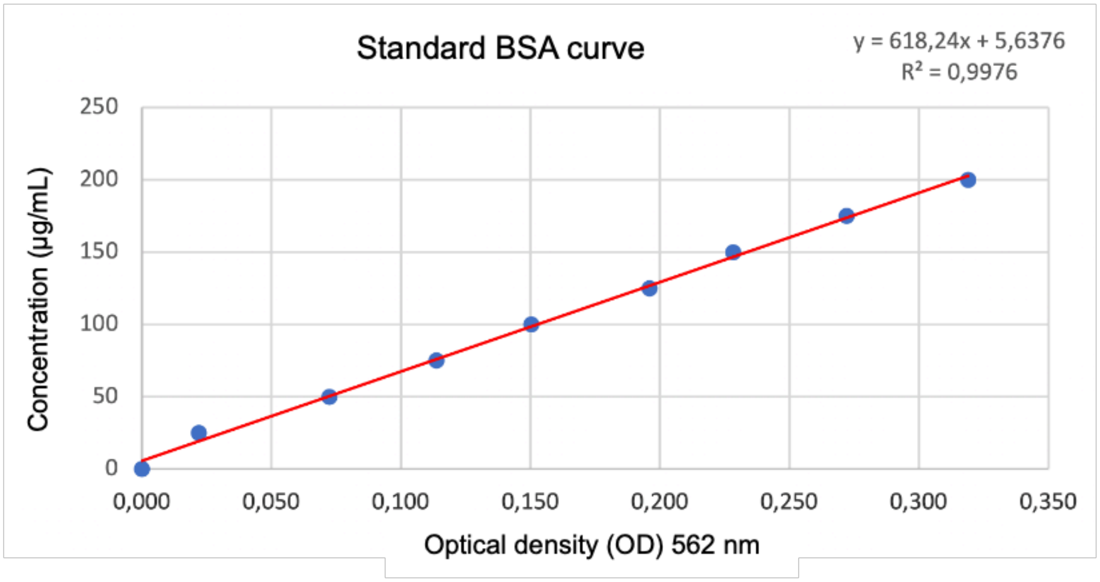

Supplementary Figure 3-1: Resulting modified BSA standard curve. Each concentration was done in triplicate and plotted as an average.

4 Supplementary Section 4: Optimization of Protocol for Extraction and Analysis of Proteins from Halite Brine Inclusions.

This new TRIzol reagent-based method for direct biomolecule extraction from halite crystals without prior dissolution step was optimized from a previous protocol from Kirkland *et al.,* 2006 for liquid haloarchaeal cultures. As described in section 3.6 of the Materials and Methods, crystals were crushed directly in TRIzol. The combined effects of the halite and the release of intracellular K^+ and Cl^- from *H. salinarum* cells upon lysis resulted in high salt concentrations in the phenol phase. Kirkland et al., showed that salts were removed in the following isopropanol, guanidine HCl and acetone steps. To ensure that no salts remained in the final protein pellets (for example combined with traces of phenol) we used two washes with 95% ethanol, 0.3 M guanidine HCl. Then guanidine HCl traces were removed using 100% ethanol wash prior to additional washes in glacial acetone (-20 °C; x2).

However, the wash steps with glacial acetone rendered protein solubilization difficult. To resolve this problem, we tested four different solubilization solutions at room temperature and 4 °C. Buffers tested included $NaHCO_3$ 1M, 0.1 % SDS ("SDS Na"); NH_4HCO_3 0.5 M, 1 % SDS ("SDS NH4"); NH_4HCO_3 0.5 M, 8 M urea ("Urea NH4"); NH_4HCO_3 0.5 M, urea 7 M, thiourea 2 M, CHAPS 4 % (w/v) ("Mix NH4"). Following solubilization and subsequent trypsin digest, LC-MS/MS (micro-injection) results (see Supplementary Figure 4-1) showed a higher number of peptides and proteins identified for urea, thiourea and CHAPS mix. However, the CHAPS buffer was ultimately eliminated due to its incapability with the BCA protein assay. Ammonium bicarbonate with 1 % SDS presented few proteins, which can be explained by partial digestion likely due to degradation of trypsin by high SDS content. Finally, while a comparison of ammonium bicarbonate with urea and sodium bicarbonate with SDS show similar results, the sodium bicarbonate buffer was ultimately chosen to avoid any interaction of ammonium ions during iTRAQ® labelling. Better results were obtained (slightly more peptides and proteins and less turbidity in sample tubes) at room temperature than at 4 ˚C.

Optimization of disulfide bonds reduction by TCEP was also performed to ensure optimal tryptic protein digestion. Two concentrations were tested as recommended by the supplier: 2 mM and 10 mM. The higher concentration resulted in fewer proteins than 2 mM concentration (data not shown), therefore 2 mM TCEP solution was used for reduction step.

Prior to LC-MS/MS injection, sample require cleaning and final desalting by solid phase extraction (SPE). We tested two commonly used cartridges: C18 and C8 (Sep-Pak Plus Short, 400 mg Sorbent). No differences were observed regarding number of peptides and proteins identified using either cartridge, and therefore C18 cartridges were selected for proteomics analyses.

The trypsin digests were analyzed by LC-MS/MS on an ultra-high-performance LC system (Ultimate 3000 RSLC, Thermo Scientific) connected to high-resolution electrospray ionization – quadrupole – time of flight (ESI-Q-TOF) mass spectrometer (Maxis II ETD, Bruker Daltonics). Separations were achieved on an Acclaim RSLC Polar Advantage II column (2.2 µm, 2.1 × 100 mm, Thermo Scientific) at a flow rate of 300 µL/min, using the following gradient of solvent A (ultra-pure water / 0.1 % formic acid) and solvent B (HPLC-MS grade acetonitrile / 0.08 % formic acid) over a total run time of 20 min linear increase from 2 % to 100 % B for 15 min, linear increase to 100 % B for 1 min, decrease to 2 % B for 1 min. The ESI-Q-TOF instrument was externally calibrated before each run using a sodium formate solution consisting of 10 mM sodium hydroxide in isopropanol / 0.2 % formic acid (1:1, v/v). Data-dependent LC-MS/MS data were acquired in positive ion mode in the mass range m/z 150 – 2200, using collision induced dissociation with collision energy calculated from

m/z and charge states. The LC-MS/MS data were treated with Data Analysis 4.4 (Bruker Daltonics) and PEAKS software as previously explained in methods section.

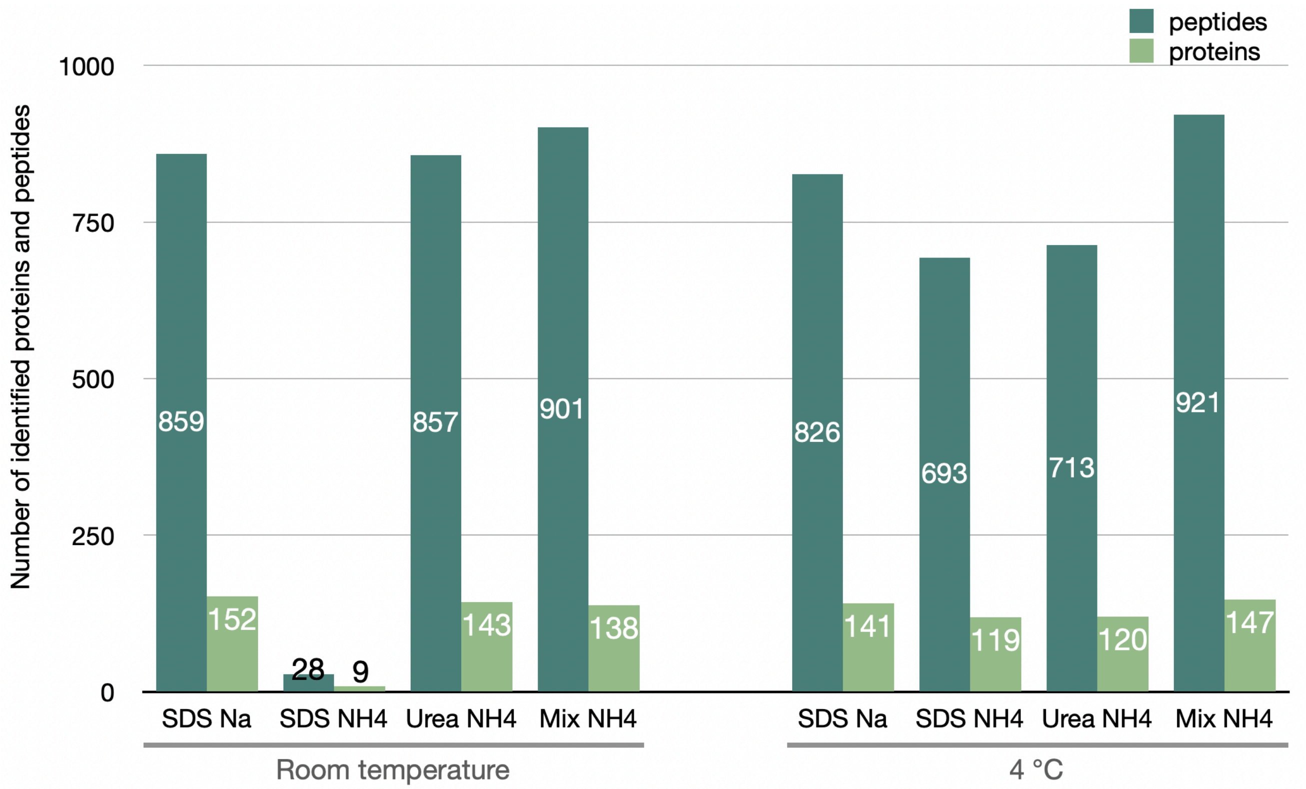

Supplementary Figure 4-1: Proteins and peptides identified by mass spectrometry for the four tested resuspension buffers: $NaHCO_3$ 1M, 0.1 % SDS (“SDS Na”); NH_4HCO_3 0.5 M, 1 % SDS (“SDS NH4”); NH_4HCO_3 0.5 M, 8 M urea (“Urea NH4”); NH_4HCO_3 0.5 M, urea 7 M, thiourea 2 M, CHAPS 4 % (w/v) (“Mix NH4”). Resuspension at room temperature and 4 °C were compared.

5 Supplementary Section 5: Comparison of Passive Baths and Active-spray Washes for Chemical Halite Surface Cleaning.

Chemical treatments for crystals surface cells and proteins removal were first tested as described in Sankaranarayanan *et al.*, 2011. Wash bath incubation times of 15 minutes completely dissolved the halite crystals, so baths were reduced to 5 min incubations. Proteins were then extracted from treated halite crystals using these passive bath washes. As seen in Suppl. Fig. 5-1, this protocol resulted in high levels of residual proteins. Instead, an active-spray procedure was tested for NaCl washes, resulting in near-total removal of proteins from halite surfaces when combined with NaOCl-NaOH-HCl treatments.

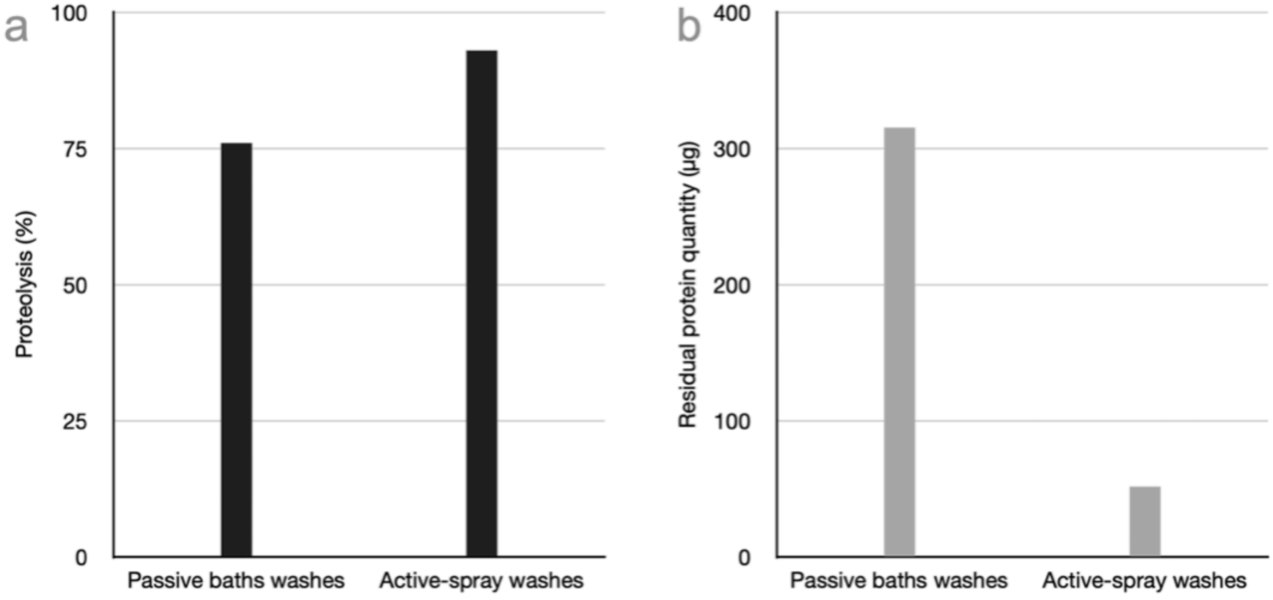

Supplementary Figure 5-1: Removal of surface-bound proteins using passive baths and active-spray washes during NaOCl-NaOH-HCl treatment on externally inoculated crystals.

6 Supplementary Section 6: Complete Proteomics Results

Complete raw data sets for proteomics analyses are provided as separate .csv files (see Supplementary Tables 6-1, 6-2, 6-3 and 6-4).

Protein identification file **Supplementary Table 6-1** give PEAKS DB results without redundancies in proteins identifiers. Sheet 1 to 4 for liquids stationary samples (corresponding to Figure 4a), sheets 5 to 8 for brine inclusion extracts (corresponding to Figure 4b), sheets 9 and 10 for total core proteins of liquids stationary and brine inclusion samples respectively (Figure 4a,b). Sheets 11 and 12 summarized specific proteins for liquids stationary and brine inclusion samples. Sheet 13 corresponds to shared core proteins between both conditions with multiple proteins identifiers and correlated descriptions.

KEGG blastKOALA matched KO identifiers given in **Supplementary Table 6-2** were subdivided as sheet 1 for shared proteins between liquid and brine inclusions, sheet 2 for specific proteins of brine inclusion extracts, sheet 3 for specific proteins of liquid stationary extracts, sheet 4 corresponding to quantified proteins showing up-regulation and sheet 5 for quantified proteins showing down-regulation from brine inclusions.

KEGG pathway search BRITE results were given in **Supplementary Table 6-3**. Results for specific proteins identified in brine inclusion extracts (sheet 1), specific proteins identified in liquids stationary extracts (sheet 2) and those shared between both conditions (sheet 3). Then results for proteins showing significative up-regulation (sheet 4) and down- regulation (sheet 5) from brine inclusions.

Supplementary Table 6-4 correspond to raw PEAKS Q quantitation results for iTRAQ sample injections for all eight samples (four replicates each liquid stationary phase extractions and halite brine inclusions extractions); first injection (replicate 1, sheet 1), second injection (replicate 2, sheet 2) and third injection (replicate 3, sheet 3) with redundancies in protein identifiers removed. Sheet 4 recapitulates the 68 proteins identified after pool of tree replicates results with multiple proteins identifiers and correlated descriptions.

Supplementary Table 6-5 summarizes proteins identified and quantified for targeted pathways. These table include identified proteins shared by the four stationary and four brine inclusion extracts, proteins partially shared by the eight samples, and proteins with significant differential expression in brine extract compared to stationary cells.

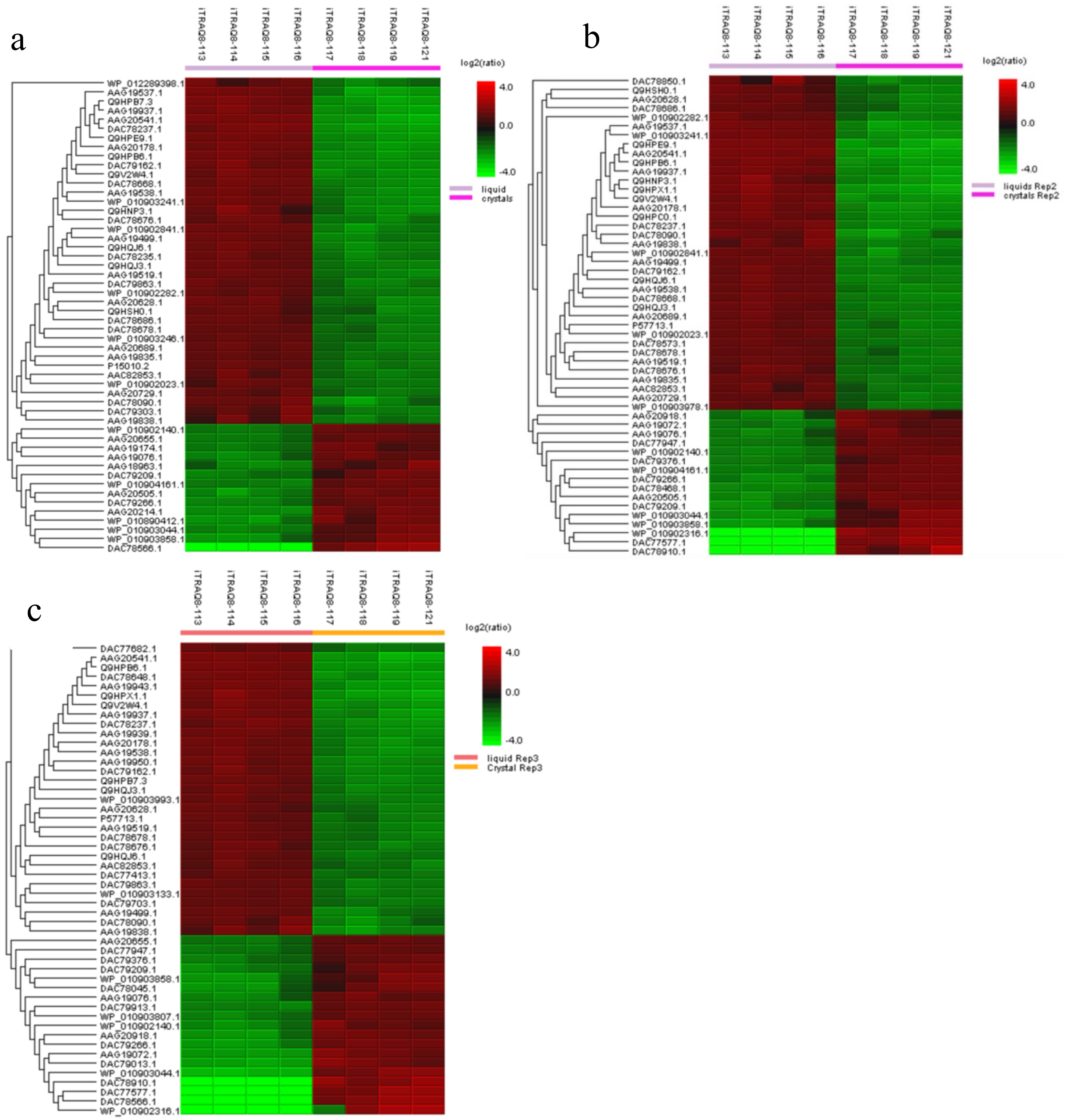

Supplementary Figure 6-1: Heatmaps for identified proteins showing differential expression in brine inclusions extracts ("crystals") compared to liquid stationary-phase cultures ("liquid") for the first (a), second (b) and third (c) injection replicates.

7 Supplementary Section 7: The Special Case of Transmembrane Peptides.

TRIzol-based protein extraction seems unsuitable for transmembrane peptides. As shown below for ArcD arginine-ornithine antiporter example (Supplementary Figure 7-1), none of the transmembrane regions of the ArcD protein were identified in this study, whereas regions extending outside the membrane were detected by LC-MS/MS.

MVEFEPRSYEDFDPEKRPSFGQ**ALLPIAGMITFLAVGIVLLGL**DAQM**PLLWGIAFTGVIARYGWGYTW**DELFDGISNS**IVMGLGAIFILFIIY**
MLIASWVDAGTIPFIMYWGLEF**LTPAVFVPLAALLSFVVATAI**GSSWTTAGSLG**IALVGIGSGLGIPAPLTAGAI**LSGVYMGDKQSPLSDTTLL
ASGVSDVDLWDHVRG**MFPNTIIVGVISLALYAVLG**LMAETGGTGGTGAEVAQIQGGLA**GTYTLSVLVLLPLVITFGLAI**KG**YPALPSLGAGV**
FSAAGVSILIQGRGFAEAWQIIYSGTGPKTGVDLVNNLLSTGG**LEGSIWVITIVFGALSIGGIL**EATGVLSVIAHNTAKAVD**SVGGATLVTALGP**
LVINALTADQYMSIVIPGMTFRDLNDEYDLDGTSLSRTLEETGTVSEPMIPWNSGGVFMASALGVP**VLSYLPYYFVGILSPILVVIM**GFTGWK
MYMKDPEESPEESADTAA

Supplementary Figure 7-1: Arginine-ornithine antiporter ArcD amino acid sequence with transmembrane peptides in blue and peptides identified in samples 1, 2 and 3 from liquid stationary-phase culture extracts in yellow.

8 Supplementary Section 8: Total RNA extractions and analysis.

The results of cell viability and proteome expression analyses of *H. salinarum* trapped within the halite crystals after two months indicated while cells were viable, proteome expression was altered after acclimation to the fluid inclusion micro-environment. In particular, the expression of ribosomal proteins was markedly reduced compared to cells from liquid cultures. To test the hypothesis that transcriptional activity was reduced in acclimated cells, we extracted RNA from the inclusions.

The objective was two-fold; first to test the TRIzol reagent method for the extraction of RNA directly from halite crystals, and second to conduct transcriptomic analyses by RNA-seq complementary to the proteomic results. Correlation between mRNA and expressed proteins would then be proof of 'cellular origin' (i.e.: produced by active cells rather than proteins preserved from inactive or dead cells) for these proteins. The advantage of a TRIzol-based extraction method is that it sequentially separates DNA, RNA, and proteins, with the potential for additional purification steps to isolate lipids as well (Podechard *et al.,* 2018). To complement the proteomics analyses presented in this study, RNA was extracted from surface cleaned, internally inoculated halite after two months of incubation at 37 ˚C, as well as stationary growth phase liquid cultures.

All RNA extraction steps were performed with RNAases-free material, solutions and glassware to avoid both RNA degradation and any organics contaminations from plastics. Total RNA for transcriptomics analyses were extracted using the TRIzol-based method described above (see Supplementary Sections 2 and 4) for protein extractions with the following modifications. To avoid RNA degradation, the TRIzol reagent mixture with pulverized halite crystals was incubated for 5 min at room temperature rather than 20 min at 65 °C as recommended by the manufacturer. The chloroform phase was then collected by centrifugation and RNA purification was perform using RNeasy MinElute cleanup columns (Qiagen). Resulting RNA were quantified using QuBit Q32857 (Invitrogen) the purity assayed using Nanodrop 2000/2000c (Thermo Scientific), and the quality was determined with the BioAnalyzer 2100 system (Agilent). Analyses were performed according manufacturer's instructions.

RNA extracted from liquid cultures was of high quality and quantity, confirming use of the TRIzol-based method for RNA extractions from *H. salinarum* cultures at 4.28 M NaCl (Lomana *et al.*, 2020). However, RNA extracted from halite fluid inclusions was degraded, with low scores for both quality and quantity. The RNA obtained from halite was therefore insufficient to allow transcriptomic analyses with RNA-seq approaches (see **Supplementary Figure 8-1, Supplementary Table 8-1**), which require at least 2 µg of RNA, with purity values >2.0 (for $OD_{260/280}$ and $OD_{260/230}$) and non-degraded RNA. The quantity of RNA extracted from internally inoculated halite sample 1 too low for accurate $OD_{260/280}$ and $OD_{260/230}$ measurements, resulting in the observed heterogeneity in the purity scores compared to the other two replicates. The RNA degradation indicate that further improvements are needed to extract RNA from halite fluids inclusions including desalting steps. However, the low RNA quantities are consistent with the reduced quantities of ribosomal proteins observed for halite inclusions extracts, suggesting low transcriptional activity in *H. salinarum* cells acclimated to fluid inclusions.

Supplementary Table 8-1: Total purified RNA quality check results after TRIzol-based extraction. Purity scores below 2.0 indicate purity unsuitable for RNAseq analyses. RNA BDL = RNA quantity below detection limit (>20 ng/mL); N/A = Not Applicable (RNA too degraded for RIN calculations, (see Supplementary Figure 8-1 for BioAnalyzer results).

	Liquid stationary cells (n=3)	Internally inoculated halite (2 months)		
		Halite 1	Halite 2	Halite 3
Halite weight (g)		1.44	2.18	1.08
RNA quantity (µg)	1092.0 ± 85.6	BDL	4.4	13.9
Purity (absorbance 260 / 280 nm)	2.1 ± 0.01	1.95	1.54	1.61
Purity (absorbance 260 / 230 nm)	2.4 ± 0.05	4.67	0.96	1.44
RNA integrity number (RIN)	9.2 ± 0.7	RNA BDL	N/A	N/A

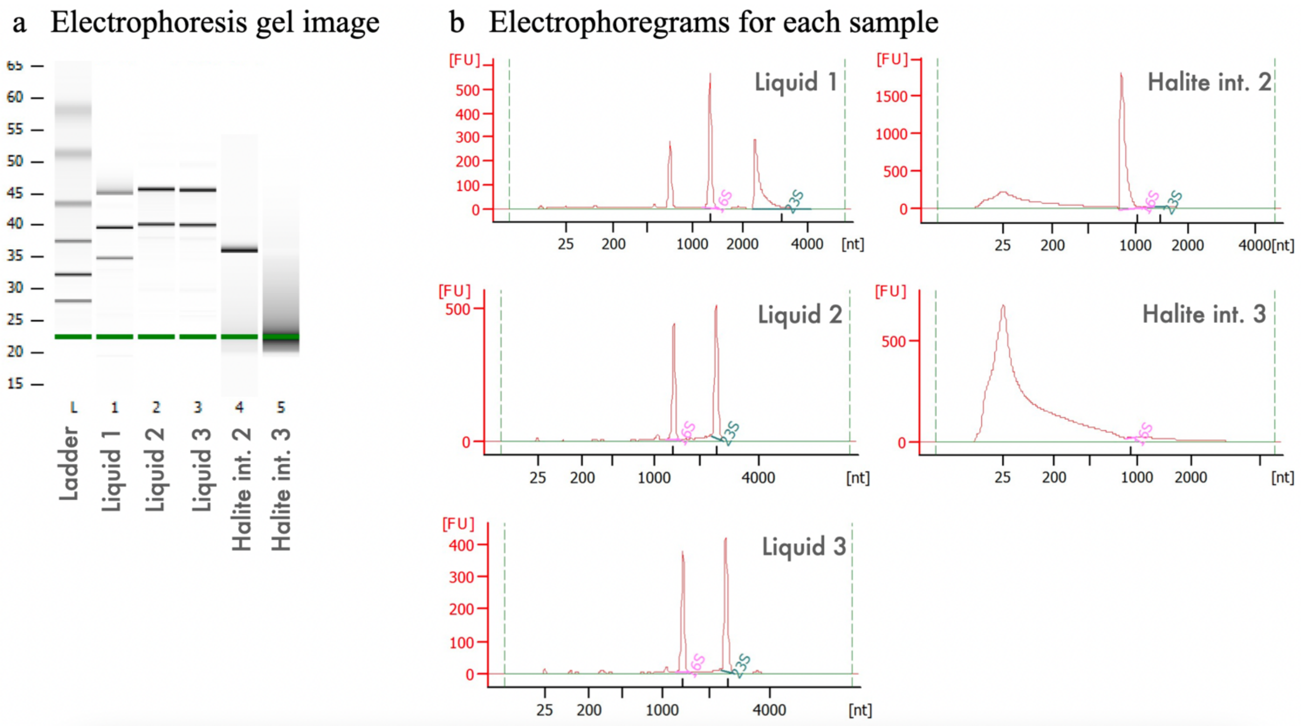

Supplementary Figure 8-1: BioAnalyzer results showing degraded RNA. (a) Electrophoresis gel image and (b) Electropherogram for each sample (FU = fluorescence units; nt = nucleotides). "Liquid" refers to RNA from liquid stationary cells, "Halite int." was used for RNA extracted from brine inclusions of halite crystals following 2 months of incubation and after surface cleaning. Numbering refers to biological replicates of liquid and halite samples. RNA quantity from 'Halite ext. 1' replicate was too low for analysis (see Supplementary Table 8-1).

9 Supplementary References

Denner, E. B. M., McGenity, T. J., Busse, H.-J., Wanner, G., Grant, W. D., Stan-Lotter, H. (1994). *Halococcus salifodinae* sp.nov., an archaeal isolate from an Austrian salt mine. *Int. J. Syst. Bacteriol.* 44, 774–780. doi: 10.1099/00207713-44-4-774.

Dombrowski, H. (1963). Bacteria from Paleozoic salt deposits. *Ann. N. Y. Acad. Sci.* 108, 453–460. doi: 10.1111/j.1749-6632.1963.tb13400.x

Gruber, C., Legat, A., Pfaffenhuemer, M., Radax, C., Weidler, G., Busse, H.-J., et al. (2004). *Halobacterium noricense* sp. nov., an archaeal isolate from a bore core of an alpine Permian salt deposit, classification of *Halobacterium* sp. NRC-1 as a strain of *H. salinarum* and emended description of *H. salinarum*. *Extremophiles* 8, 431–439. doi: 10.1007/s00792-004-0403-6.

Gramain, A., Díaz, G. C., Demergasso, C., Lowenstein, T. K., and McGenity, T. J. (2011). Archaeal diversity along a subterranean salt core from the Salar Grande (Chile). *Environ. Microbiol.* 13, 2105–2121. doi: 10.1111/j.1462-2920.2011.02435.x.

Norton, C. F., and Grant, W. D. (1988). Survival of Halobacteria Within Fluid Inclusions in Salt Crystals. *J. Gen. Microbiol.*, 1365–1373. doi: 10.1099/00221287-134-5-1365.

Norton, C. F., McGenity, T. J., and Grant, W. D. (1993). Archaeal halophiles (halobacteria) from two British salt mines. *Microbiology*, v. 139, p. 1077–1081, doi: 10.1099/00221287-139-5-1077.

Radax, C., Gruber, C., and Stan-Lotter, H. (2001). Novel haloarchaeal ^{16}S rRNA gene sequences from Alpine Permo-Triassic rock salt. *Extremophiles* 5, 221–228. doi: 10.1007/s007920100192.

Rosenzweig, W. D., Peterson, J., Woish, J., and Vreeland, R. H. (2000). Development of a Protocol to Retrieve Microorganisms from Ancient Salt Crystals. *Geomicrobiol J.* 17, 185–192. doi: 10.1080/01490450050121152.

Sankaranarayanan, K., Timofeeff, M. N., Spathis, R., Lowenstein, T. K., and Lum, J. K. (2011). Ancient Microbes from Halite Fluid Inclusions: Optimized Surface Sterilization and DNA Extraction. *PloS One* 6, e20683. doi: 10.1371/journal.pone.0020683.

Stan-Lotter H., Sulzner M., Egelseer E., Norton C. F., and Hochstein, L. I. (1993) Comparison of membrane ATPases from extreme halophiles isolated from ancient salt deposits. *Orig. Life Evol. Biosph.* 23, 53–64. doi: 10.1007/s007920100192.
